# Decreased Phototoxic Effects of TiO₂ Nanoparticles in Consortium of Bacterial Isolates from Domestic Waste Water

**DOI:** 10.1371/journal.pone.0141301

**Published:** 2015-10-23

**Authors:** Ankita Mathur, Jyoti Kumari, Abhinav Parashar, Lavanya T., N. Chandrasekaran, Amitava Mukherjee

**Affiliations:** Centre for Nanobiotechnology, VIT University, Vellore, India; Indian Institute of Toxicology Research, INDIA

## Abstract

This study is aimed to explore the toxicity of TiO_2_ nanoparticles at low concentrations (0.25, 0.50 & 1.00 μg/ml); on five bacterial isolates and their consortium in waste water medium both in dark and UVA conditions. To critically examine the toxic effects of nanoparticles and the response mechanism(s) offered by microbes, several aspects were monitored *viz*. cell viability, ROS generation, SOD activity, membrane permeability, EPS release and biofilm formation. A dose and time dependent loss in viability was observed for treated isolates and the consortium. At the highest dose, after 24h, oxidative stress was examined which conclusively showed more ROS generation & cell permeability and less SOD activity in single isolates as compared to the consortium. As a defense mechanism, EPS release was enhanced in case of the consortium against the single isolates, and was observed to be dose dependent. Similar results were noticed for biofilm formation, which substantially increased at highest dose of nanoparticle exposure. Concluding, the consortium showed more resistance against the toxic effects of the TiO_2_ nanoparticles compared to the individual isolates.

## Introduction

Nano-scale particles are found naturally in the environment. TiO_2_-NPs are an important component of paints, sunscreens, food additives and coloring compounds and are also used for ground water remediation [1]. TiO_2_-NPs has the highest production rate estimated around 5000 tonns till 2010 and is predicted to increase up to 2.5 million tons by 2025 [2]. The International Agency for Research on Cancer (IARC) has rated TiO_2_ as the most carcinogenic compound (Group 2B) for humans [3]. The permissible and recommended exposure levels of TiO_2_-NPs have been assessed to 1.5 and 1 mg m^-3^ respectively [4]. TiO_2_-NPs in surface water is reported to be 1 μg/ml [5]. Waste water is considered as a major entry point for TiO_2_ into the aquatic system. The concentration of Ti^4+^ ions has been estimated to be 181–1233 μg/ml in waste water treatment plants [6, 7]. The dynamic processes such as the discharge of nanaoparticles, through product degradation, waste water effluents, sludge and other routes affect the fate and nanoparticles toxicity mechanism in environment and hence are a major concern of study [8].

Secondary treatment of waste water is primarily processed by various bacterial colonies. The potential of bacteria to reduce organics in sewage is affected by several factors like pH, temperature, dissolved oxygen, agitation, etc. The engineered nanoparticles directly affect the ecological cycle and all strata of food chain. The most affected organisms are bacteria as they are ubiquitous and are important in nutrient cycling. These are generally used as model organisms for ecotoxicological studies. Nanoparticles have the capacity to change the pH and dissolved oxygen in a medium via redox activities. TiO_2_-NPs are toxic to all kinds of bacteria, although, showing lesser effects on gram positives [9–11]. There are few approaches that have been focused while studying nanotoxicity while examining membrane integrity, changes in post exposure bacterial morphology and uptake of nanoparticle by the cells [12–14].

Extracellular Polymeric Substances (EPS) are released generally under stressed conditions as a defense mechanism to assist the bacterial colonies to form aggregates and develop biofilms. Nanoparticles impart an oxidative stress which signals the colonies to develop such protective environment across the cells, as also demonstrated by reports from NASA (Astrobiology). Biofilms are multicellular communities residing in hydrated complex structure of EPS [15]. The structural support provided by EPS to biofilms acts as a resistance to physical damage. The attachment of TiO_2_-NPs to the surface of EPS matrix retains them immobilized thereby minimizing its interaction with the biofilm. There are a few reports depicting the induction of biofilm formation as a result of inflammatory response to TiO_2_-NPs [16–18]. There are some reports depicting the biofilm formation with single species as well as multispecies important to waste water treatment process [19].

Our previous study [20] with TiO_2_-NPs mainly focused on the types of ROS generation under UVA, visible light and dark conditions with single dominant waste water bacterial isolate. The present work rather deals with the comparative toxic effects of TiO_2_-NPs on five dominant individual waste water isolates and their consortium in test medium. The most dominant five bacterial isolates selected for this study are of diverse characteristics. *Exiguobacterium acetylicum* (E.A.), *Exiguobacterium indicum* (E.I.) are Gram positive.,facultative anaerobes. *Pseudomonas nitroreducans* (P.N.), *Brevundimonas diminuta* (B.D.) and *Bacillus flexus* (B.F.) are aerobic. Prior two being Gram negative while the latter is Gram variable. *Exiguobacterium acetylicum* is a rhizospheric, rod shaped bacteria and has been recognized as PGPB (Plant Growth Promoting Bacteria). *Exiguobacterium indicum* is rod shaped, motile, non-spore forming, alkaliphilic bacterium. Both of these bacteria come under the genus *Exiguobacterium* and are also found in waste water. *Pseudomonas nitroreducens* is an aerobic soil bacterium isolated from waste water. It has the capability to synthesize polyester from fatty acids. It has been also been noticed that the bacteria has the ability to metabolize PHA (polyhydroxyalkanoates), PHB (polyhydroxybutyrate) and azo dyes. *Brevundimonas diminuta* and *Bacillus flexus* are generally found in the waste water effluent and in the downstreaming process of waste water treatment process.[21–24].

A systematically unique approach to gain holistic insights towards the phototoxic effects on both single isolates and the consortium was explored in filtered and consecutively sterilized waste water medium. The natural organic colloides present in the test medium was maintained throughout the study as it mimics the real environment of the waste water. The test medium already contained 0.77 μg/ml Ti^4+^ ions. We hypothesize that the tolerance againt the (photo)toxic effects of TiO_2_-NPs would be greater in consortium due to synergistic effects of single dominant bacterial species. It is therefore expected to observe an increase in EPS release and biofilm deposition. The consortium was considered for this study to imitate the associations of bacterial colony in the source plant.

## Materials and Methods

### Materials Procured

Dyes: Propidium iodide, acridine orange and 2', 7-dichlorofluorescin diacetate were procured from Sigma-Aldrich (India).

Chemicals: Sodium pyruvate, riboflavin, nitroblue tetrazolium chloride, dry titanium (IV) oxide anatase nanoparticles (< 25 nm) were obtained from Sigma-Aldrich and ethylene diamine tetra acetic acid (EDTA), Tris HCl was from Himedia.

Media: Nutrient Broth and nutrient agar was obtained from Himedia

All the chemicals used throughout the study were of analytical grade.

### Physicochemical characterization of waste water

The waste water was collected from VIT sewage treatment plant (secondary treated tank that involves removal of organic matter after primary treatment, and is usually performed by indigenous bacteria) in sterile polypropylene bottles and stored at 4°C till further analysis. The waste water used for the study had a pH of 7.7, dissolved oxygen at 5.41 mg/L, conductance of 165 ± 0.9 mS and total organic carbon at 18.1 ±0.8 μg/ml, total dissolved salt of 2.00± 0.02 μg/ml. Primary filtration was carried out with the help of 20 μm sieve to remove the suspended solid particles. The filtered sample was further filtered through Whatman No.1 filter paper and secondary filtration with the help of 0.22 μm syringe filter to avoid the intervention of colloidal particles. This filtered and consecutively sterilized (autoclaving at 121°C, 15 psi, 15 min) waste water medium was used for the experiments throughout this study, also referred as treated waste water here-forth.

### Stability of nanoparticles in waste water medium by Dynamic Light Scattering (DLS)

A stock suspension of 100 μg/ml TiO_2_-NPs was prepared in Cascada™ ultrapure biowater (Pall Corporation) and subjected to ultrasonication at 130 W for 10 min (Ultrasonics, USA). A working concentration of 0.25, 0.5 and 1 μg/ml of TiO_2_-NPs was prepared in treated waste water by addition of appropriate amount from stock suspension. Stability of these particles was checked at 0, 6 and 12 h under dark and UVA condition. The effective diameter was measured using particle size analyser (Brookhaven Instruments Corporation, USA).

### Isolation and identification of microbes

The collected waste water was subjected to standard dilution in aseptic conditions. Primary isolation was carried out by streaking the sample on the surface of the nutrient agar plate. The plates were then further incubated at 37°C for 24 h. Single isolated colonies were selected, and sub cultured on the nutrient agar plate. This process was repeated untill pure culture was obtained. The dominant bacterial isolates were selected for the study. According to the standard methods, morphology and Gram tests were accomplished with the assistance of microbial taxonomy [25]. The genomic DNA was extracted employing phenol chloroform and fluorescent dye terminator method (Big dye terminator cycle sequencing kit, ABI Prism 3.1).[26] The sequences were examined with BLAST and aligned with help of CLUSTAL W facilitating neighbor joining method and phylogenetic tree was constructed. The waste water isolate exhibited 99% similarity in BLAST. The five bacterial isolates identified were *Exiguobacterium acetylicum* (VITWW1), *Pseudomonas nitroreducens* (VITWW2), *Bacillus flexus* (VITWW3), *Brevundimonas diminuta* (VITWW4) *Exiguobacterium indicum* (VITWW5). The 16SrRNA sequences were submitted to Genbank and the accession ID was obtained to be as (KJ146070) for *Exiguobacterium acetylicum*, (KT272871) for *Exiguobacterium indicum*, (KT272873) for *Bacillus flexus*, (KT272872) for *Brevundimonas diminuta* and (KJ146071) for *Pseudomonas nitroreducens*.

No specific authorization was necessary for collecting samples for the study as the waste water plant is situated inside the University campus. The experiments involved were carried out under the confinements of the laboratory and due care was exercised not to contaminate or disrupt the ecosystem.

### Synergistic and antagonistic studies following the development of the consortium

To assess the effects of TiO_2_-NPs on waste water isolates, five bacterial isolates and their consortium was selected for the study. Usually, in the natural environmental conditions, the bacterial cells aggregate with each other and form the consortium. The selection of the consortium for the study was more appropriate as these are divergent and may exhibit less toxic effect than individual homogenous isolates. For the development of the consortium, five waste water bacterial isolates (*Exiguobacterium acetylicum*, *Exiguobacterium indicum*, *Pseudomonas nitroreducens*, *Bacillus flexus* and *Brevudimonas diminuta*) were assayed for synergistic and antagonistic effects. One isolate was taken and allowed to grow in nutrient broth for 8 h. After the incubation period, 100 μl of one of the culture broth was poured on the surface of nutrient agar and a loop full of culture from second isolate was streaked on the nutrient agar plate. The plates were incubated at 37°C for 24 h and were further examined. All the five strains were checked accordingly, and the zone of inhibition was found to be missing, thereby depicting the absence of competitive inhibition[27].

### Cell viability assessment

The waste water bacterial isolates and their consortium were selected for cell viability examination upon incorporation of TiO_2_ NPs. All the five isolates were grown individually and in the consortium,in nutrient broth till the exponential phase was reached. The culture was then spun at 7000g for 10 mins. The harvested cells were further washed twice with the treated waste water to remove the components of growth media. A fixed cell count of 5 × 10^8^ cells were maintained throughout the experiments. Standard plate count assay was performed thereafter. Individual bacteria and the consortium were treated with three concentrations of TiO_2_-NPs dispersions (0.25, 0.5 and 1 μg/ml). All the assays were performed under dark condition (no irradiation) by covering the beakers with opaque sheets and UVA (350 nm, 18 watts at 1mW/cm^2^). The experiments were conducted at 37°C for 24 h at 120 rpm in a shaking incubator (Scigenics Biotech, Orbiteck).

### Framework of the experiment

To evaluate the toxic effect of TiO_2_-NPs on individual waste water isolate and the consortium, the cell viability assay was performed. The control cells at 0^th^ hour were considered to be 100% viable. A decrease in the percentage viability of the treated samples was calculated with respect to the control. Standard plate count assay on nutrient agar was followed. The data presented are an average of triplicates.

### Confocal Laser Scanning Microscopy (CLSM)

CLSM aims to check the three-dimensional structure of the organism [28]. Control and the consortium cells were interacted with 1 μg/ml of TiO_2_-NPs for 6h and were stained with 500μl of propidium iodide and acridine orange for 10 min. Stained consortium cells were washed thrice with 2X saline sodium citrate (SSC) buffer. The cells were observed with confocal laser microscope (Zeiss LSM 5 10 META Confocal System, Germany) with an excitation filter LB 543 nm and emission filter BP 565–615 nm.

### Oxidative stress assessment

#### Assessment of Reactive Oxygen Species (ROS)

To understand the possible toxicity mechanism of TiO_2_-NPs towards waste water individual isolates, as well as the consortium, the production of ROS was measured. The intracellular ROS activity was measured by non-polar fluorescence probe 2'-7' dichlor-fluorescin diacetate (DCFH-DA) oxidation to its hydrophilic derivative DCFH. The activity of the dye in the presence of esterases and ROS gives a green fluorescent DCF. 5 ml of cell suspension was incubated with DCFH-DA with a final concentration of 100 μM at 37°C for 30 min. ROS generation was measured in control and NP-treated cells for a period of 24 h following a standard protocol [29]. To estimate the autofluorescence activity of dye, a negative control containing NPs without bacterial cells was also analyzed. Fluorescence was measured using fluorescence spectrophotometer (Model G9800A, Cary Eclipse fluorescence spectrophotometer, Agilent Technologies, USA) with excitation and emission of 485 and 530 nm respectively.

#### Determination of Superoxide Dismutase (SOD) activity

SOD enzymes can detoxify superoxide anions, with the generation of free radicals [30]. The method is based on the ability of the enzyme to inhibit the reduction of nitroblue tetrazolium (NBT) by superoxide, generated by photo reduced riboflavin and oxygen [31]. The control and interacted cells with 1 μg/ml of TiO_2_-NPs were centrifuged at 7000 g for 10 min and the supernatant was collected. To the supernatant 0.1 M EDTA and 1.5 mM NBT was added. It was followed with the addition of riboflavin and thereafter a subsequent incubation for 30 min at room temperature. The activity of SOD was determined by the absorbance at 530 nm using UV–Vis spectrophotometer (U-2910 spectrophotometer, HITACHI).

#### Assessment of cell membrane integrity

The integrity of the cell membrane was evaluated by the amount of lactate dehydrogenase enzyme (LDH). The level of extracellular LDH acts as an oxidative stress marker and also an indicator of membrane permeability. The bacterial cells were interacted with the NPs for 24 h and the suspension was centrifuged at 7000 g for 10 min. The supernatant was quantified for LDH level following the standard protocol [32]. In 100 μl of supernatant, an equal volume of 30 mM sodium pyruvate and 2.8 ml of 200 mM Tris HCl was added. The decrease in the absorbance was checked at 340 nm with respect to control using UV-Vis Spectrophotometer (UV-Vis spectrophotometer 2201, Systronics).

### Internalization of NPs with help of Transmission Electron Microscopy (TEM)

The ultrastructural changes in the consortium were observed by transmission electron microscopy. The consortium for all the five isolates from waste water was made; cells were interacted with 1 μg/ml of TiO_2_-NPs for 6 h. Further, control and treated cells were centrifuged at 7000g for 10 min. The pellet was collected and washed with treated waste water. Thin sections of cells were placed on copper grids to analyze the changes in the control and treated samples of the consortium through TEM (Philips CM 12, Transmission Electron Microscope, Netherlands).

### Extraction and Quantification of Extracellular Polymeric Substances (EPS)

EPS are the complex organic high molecular weight of compounds that are released during cell lysis. The EPS production of individual bacterial isolates and their consortium was analyzed. Loop full cultures of individual and the consortium bacterial cells were grown in 100 ml of nutrient broth. The cells were incubated for 37°C for 24h at 120 rpm in a shaking incubator (Scigenics Biotech, Orbiteck). The cultures were then centrifuged at 10,000g for 10 min and the supernatant was harvested. To the collected supernatant, an equal amount of ethanol was added and was retained for overnight precipitation at 4°C. The suspended mixture obtained was centrifuged at 10,000g for 30 min and pellet was collected. The pellet was harvested and washed twice with ethanol and dialyzed against distilled water. The EPS collected was estimated with the help of phenol sulphuric method [33].

### Surface chemical studies of EPS

#### FT-IR spectral analysis

The major structural changes in purified EPS were detected using Fourier transform infrared (FT-IR) spectroscopy. The FT-IR peaks were checked for both control and interacted cells with 1 μg/ml of TiO_2_-NPs under UVA and dark condition. 5 ml of suspension was centrifuged at 10,000g for 10 min. The collected pellet was washed twice with (1x PBS) and lyophilized to remove moisture. The dried cells were then subjected to FT-IR and KBr pellet was used as a reference [34].

#### Zeta Potential measurement of EPS of the consortium

The electrophoretic mobility and the surface charge of the bacterial cells suspension plays an important role in estimating the interaction of bacteria and NPS. The bacterial EPS were treated with 1 μg/ml of TiO_2_-NPs for a period of 24 h under UVA and dark condition. The control and treated EPS were analysed for the measurement of zeta potential with the help of zeta analyser (SZ100, Horiba, Japan) [35].

### Biofilm Formation Assay

Each individual isolates and the consortium were grown for 24 h, 37°C at 120 rpm in a shaking incubator (Scigenics Biotech, Orbitech). Overnight cultures of the individual isolates and the consortium were diluted in the ratio of 1:100 in the waste water matrix. In 96 well microtitre plates, 100 μl of the bacterial suspension treated with 1 μg/ml of TiO_2_-NPs under UVA and dark condition was incubated at 37°C for 24 h in a static condition. The control (bacterial suspension without nanoparticles) and treated waste water without nanoparticles were also included in 96 well microtitre plates. Further, the 96 well microtitre plates were washed with sterile distilled water to remove the non- attached cells and were kept drying for 1 h. The dried microtitre plates were washed with 1% crystal violet for 30 min at room temperature. The remaining crystal violet was eluted with 95% ethanol solution. The retained biofilm biomass was determined by measuring absorbance at 590 nm. The assay was conducted in triplicates, and the values were recorded after subtracting the background absorbance of the wells containing NPs [16, 36].

#### Scanning Electron Microscopy of Biofilm

Scanning electron microscopy for biofilm was performed. The bacterial consortium control (absence of nanoparticles) and treated cells with 1 μg/ml of TiO_2_-NPs in waste water medium was grown in 24 well microtitre plates and incubated at 37°C for 24 h. The samples were washed with 1x PBS after 24 h incubation and was fixed with 2.5% glutaraldehyde. The fixation step was further followed by dehydration steps with 20, 40, 60, 80 and 100% ethanol and dried overnight. The glass slides were fixed on the specimen mount with the help of carbon tape. To avoid surface charge interference, gold sputtering was executed in an argon atmosphere. Morphological analysis and surface elemental composition of the biofilm was performed with the help of SEM-EDX (S-400, HITACHI and Tokyo, Japan JEOL JSM-5510) [16].

## Statistical analysis

The experiments were conducted in triplicates and represented as mean ± standard error. Toxicity evaluation was estimated with the help of one way ANOVA followed by Dunnett Post Hoc with p< 0.05. The results for LDH, SOD and ROS, was analysed with the help of two-way ANOVA followed by Bonferroni Posttest. However the graphs were prepared in Microsoft excel and the data was analysed using Graph Pad Version 5.

## Results

### Stability of TiO_2_-NPs in waste water medium

**Fig 1:**
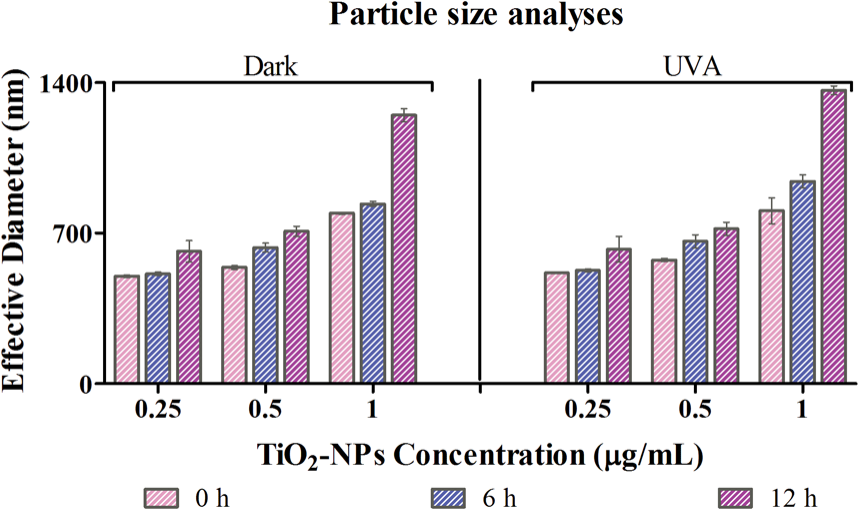
Stability of TiO_2_ NPs: With dynamic light scattering technique, size of the nanoparticles in waste water medium was analyzed. Values reported are at 0h, 6h and 12 h under dark and UVA conditions (350 nm, 18w and intensity of 1mW/cm^2^) for 0.25, 0.5 and 1 μg/ml of TiO_2_ NPs.

The stability of TiO_2_-NPs was studied in exclusively filtered and consecutively sterilized waste water medium at 0, 6 and 12 h (Fig 1). The stability was examined at three different concentration of NPs *viz*. 0.25 μg/ml, 0.50 μg/ml and 1.00 μg/ml; under both dark and UVA conditions.

Dark condition: The effective diameter of TiO_2_-NPs at 0.25 μg/ml concentration in treated waste water medium was estimated to be 500 ± 5.6, 510.2 ± 8.1 and 615.2 ± 50.3 nm at 0, 6 and 12 h respectively. Significant change in stability and hence the size of the native state of TiO_2_-NPs is observed, i.e., from initial size of < 25 nm of nanopowder to > 500 nm immediately on dispersion in waste water media. This is undoubtedly a result of aggregation of these nanoparticles. The increment in the effective diameter of the NPs at a concentration of 0.5 μg/ml was observed as 540 ± 8.3, 632.7 ± 21 and 708.5 ± 23.4 nm respectively at 0, 6 and 12 h. Whereas, at 1 μg/ml concentration, it was 792.5 ± 5.3, 836 ± 12.5 and 1249 ± 30 nm at 0, 6 and 12 h respectively.

UVA irradiated condition: At 0.25 μg/ml TiO_2_-NPs concentration, the effective diameter in this treated waste water medium was assessed to be 514 ± 3.2, 574.9 ± 7.9 and 804.4 ± 60 nm at 0, 6 and 12 h respectively. Similarly, for the suspensions with 0.5 μg/ml of TiO_2_ NPs, it was found to be 526.8 ± 6.8, 661.9 ± 30.2 and 941.7 ± 30.3 nm. The effective diameter of the particles at 1 μg/ml of TiO_2_-NPs was augmented to 625.24 ± 49, 720.26 ± 10.7 and 1364 ± 10 nm at 0, 6 and 12 h time interval respectively.

Noticeable change in the diameter is seen between 6^th^ to 12^th^ h of incubation. In addition, a marked difference between the sizes is observed only when the concentration is raised to 1 μg/ml. The effective diameter was statistically significant (p < 0.05) at 6 and 12 h time intervals. Our previous studies showed no significant chemical dissolution of TiO_2_-NPs in treated waste water medium, under dark and UVA conditions of exposure. Furthermore, the presence of Ti^4+^ ions in the procured waste water, did not affect the chemical dissolution of TiO_2_-NPs [20].

### Cell viability of individual isolates and the consortium from waste water

To assess nanoparticle toxicity, cell survival upon nanoparticle exposure was measured using colony forming unit under both dark and in UVA conditions. To estimate the toxic effect of TiO_2_-NPs on waste water isolates and their consortium, standard plate count assay was done. An exposure and concentration dependent reduction in viability was observed.

**Fig 2:**
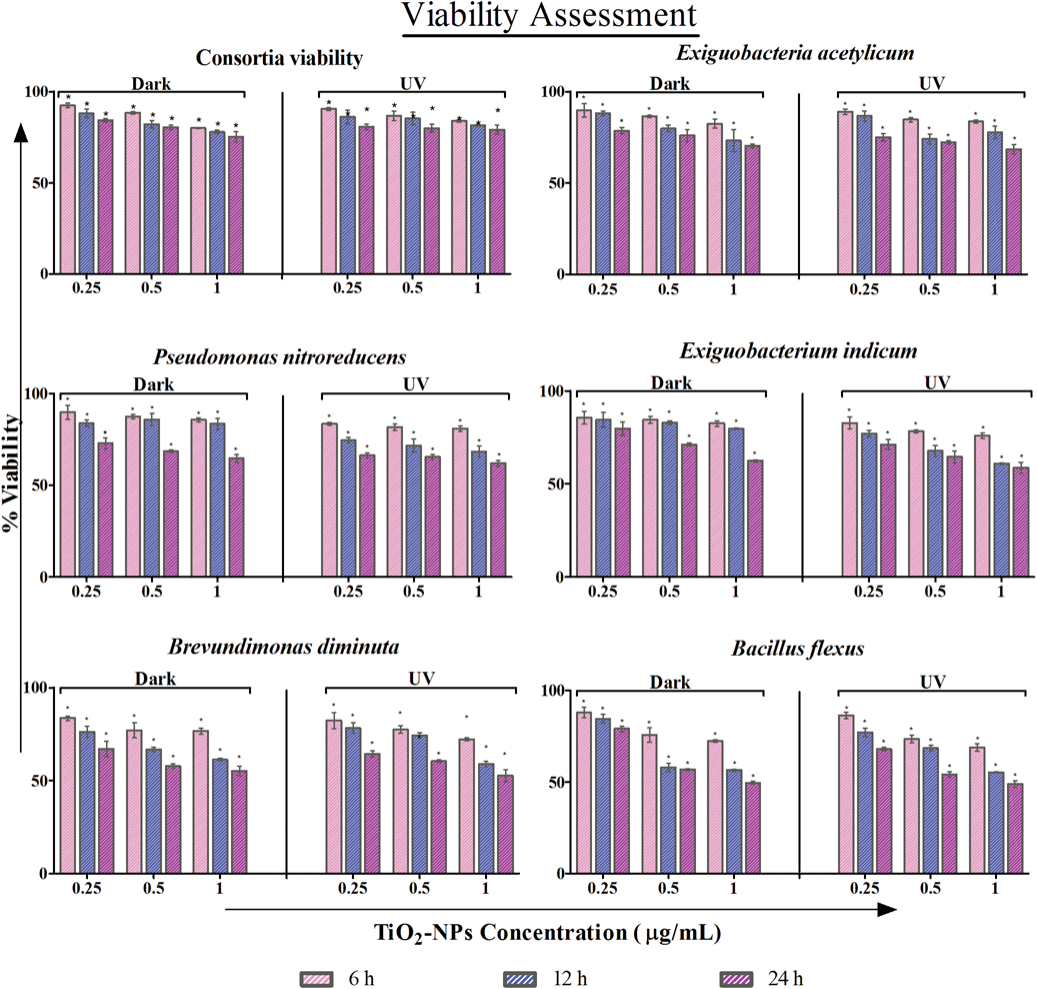
Assessment of cell viability: The percentage reduction in viability of the consortium, *Exiguobacteria acetylicum*, *Pseudomonas nitoreducens*, *Exiguobacterium indicum*, *Brevundimonas diminuta and Bacillus flexus* under dark and UVA condition at 0.25, 0.5 and 1 μg/ml of TiO_2_-NPs (n = 3). Level of significance is represented with ‘*’ between treated cell with respect to control cell under dark and UVA conditions.

Under dark: As expected, the individual cells were more prone to TiO_2_-NPs exposure. *B*. *flexus* showed least viability among the individual isolates and the consortium. And, the effect on the consortium was least. Comparing all the concentrations, 1 μg/ml had the most prolific toxic effects on viability. The percentage viability at 24 h for 1 μg/ml of TiO_2_-NPs treated cells under dark condition was observed to be 75.18 ± 2.9, 70.3 ± 2.72, 64.56 ± 4.05, 62.3 ± 3.0, 52.5 ± 3.21 and 48.9 ± 1.8% for the consortium, *E*. *acetylicum*, *P*. *nitoreducens*, *E*. *indicum*, *B*. *diminuta* and *B*. *flexus* respectively (Fig 2).

Under UVA: The profile pattern for loss in viability was similar as seen in dark condition. For NPs concentration of 1 μg/ml, viability was found to be 83.9 ± 2.5, 68.5 ± 2.72, 61.9 ± 1.7, 58.6 ± 0.66, 55.1 ± 2.5 and 49.5 ± 0.88% for the consortium, *E*. *acetylicum*, *P*. *nitoreducens*, *E*. *indicum*, *B*. *diminuta* and *B*. *flexus* respectively (Fig 2). A significant (p < 0.05) percentage reduction in viability of treated samples to control was observed with respect to exposure time (dark & UVA) and concentration. The sensitivity of TiO_2_-NPs towards the consortium was found to be less as compared to the individual waste water isolates. The reduction in viability was determined to be statistically significant (p < 0.05) for the individual isolates as compared to the consortium.

To ascertain the viability of the consortium of cells and to get topological and surface profiling evaluation, confocal laser scanning microscopy (CLSM) was performed (S1A and S1B Fig). Cells with complete integrity do not take up any of the dye and therefore remain unstained. Both the dyes used are nucleic acid selective fluorescent dyes. On one hand, acridine orange is cell permeable, therefore stains the viable cells giving a green fluorescence; while propidium iodide is a cell non-permeable dye, and hence stains the dead cells imparting a red fluorescence. Again, just the consortium of cells was chosen for this analysis. A clear differentiation between live and dead consortium cells was hence established. Treatment with TiO_2_-NPs effectively caused cell damage, which eventually affected the staining with propidium idodide seen as red in the CLSM image.

### Oxidative Stress Assessment

#### Assessment of Reactive Oxygen Species (ROS)

**Fig 3:**
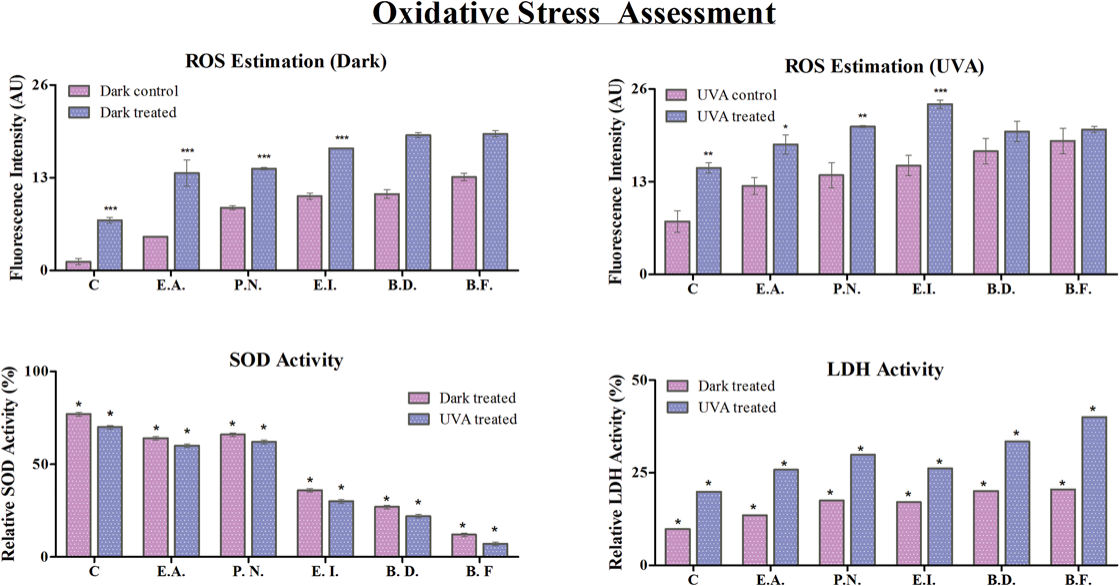
Oxidative stress analysis: Reactive oxygen species (ROS), relative SOD activity (%) and relative LDH activity at 1 μg/ml of TiO_2_-NPs treated cells under dark and UVA condition with respect to control is shown. Significant difference between control and treated cells is represented by ‘*’.

It is known that TiO_2_-NPs generate ROS from water, under UVA irradiated conditions and/or in presence of redox active molecules. ROS is a principal indicator of oxidative stress, and its analysis helps to assess the toxic effect of nanoparticles [37]. To confirm the toxicological role of TiO_2_ NPs, intracellular ROS level was checked and the corresponding fluorescence intensity of 2′, 7′-dichlorofluorescin is depicted in Fig 3.

Under dark: The ROS levels for cells without treatment were found to be least in the consortium, whereas, *B*. *flexus* showed highest amount of ROS. There was a substantial increase in ROS generation after TiO_2_-NPs treatment in all the cells under study. Since the control for the consortium showed minimal amount of ROS under dark, the percentage increase of the same was much more profound (~ 6X increase) as compared to other treated single isolates (~3X, 1.6X, 1.6X, 1.7X and 1.5X increase for *E*. *acetylicum*, *P*. *nitroreducans*, *E*. *indicum*, *B*. *diminuta* and *B*. *flexus* respectively).

With UVA exposure, the level of ROS was elevated for all the control cells, thereby indicating the photo-activation effects of UVA upon bacterial cells. But, the consortium still showed the least amount of ROS accumulation and *B*. *flexus* still had maximum ROS concentration for control cells, as observed under dark. Increase in ROS generation was, once again, maximum in the consortium (~ 2X increase) as compared to single isolates (~ 1.5X, 1.5X, 1.6X, 1.2X and 1.1X respectively for the consortium, *E*. *acetylicum*, *P*. *nitoreducens*, *E*. *indicum*, *B*. *diminuta and B*. *flexus*). *E*. *indicum*, thereby showed maximum ROS generation among the treated cells.

The ROS level was estimated to be higher for individual isolates as compared to the consortium in all cases. TiO_2_-NPs exhibited a significant (p < 0.05) increase in ROS level for the individual isolates as well as the consortium as compared to the control in both dark and UVA treated conditions. The individual isolates exhibited higher ROS production as compared to the consortium. Overall, ROS generation was least affected in the consortium under dark by TiO_2_-NPs introduction. The ROS concentration was estimated to be significantly higher (p < 0.05) under UVA condition as compared to the dark [38]

#### Determination of superoxide dismutase activity (SOD activity)

To investigate the antioxidant activity of the cells, SOD assay was evaluated in individual isolates and their consortium (Fig 3) with (treated) and without (control) exposure to 1 μg/ml of TiO_2_ NPs. Assessing SOD activity enlightens with the cells’ ability to cope up oxidative stress. Therefore, higher activity pertains to better oxidative stress management and hence better survival prospects.

Under dark: The relative SOD activity in treated cells was estimated to be 77±0.9, 64±0.96, 66±0.97, 36±0.95, 27±0.97 and 12±0.98% with respect to control for the consortium, *E*. *acetylicum*, *P*. *nitoreducens*, *E*. *indicum*, *B*. *diminuta and B*. *flexus* respectively. Clearly, SOD activity is hampered with the introduction of TiO_2_ NPs. The least affected was the consortium and the most was again *B*. *flexus*. Among the individual isolates, *P*. *nitoreducens* showed to have best SOD activity.

Under UVA condition: The relative SOD activity with the introduction of TiO_2_-NPs was approximated to be 70± 0.95, 60± 0.98, 62± 0.99, 30±0.98, 22±0.97 and 7 ± 0.99% for the consortium, *E*. *acetylicum*, *P*. *nitoreducens*, *E*. *indicum*, *B*. *diminuta and B*. *flexus* respectively. The consortiums of cells were able to retain maximum SOD activity and the maximum activity was lost for *B*. *flexus*. In UVA irradiated condition too, *P*. *nitoreducens* had the maximum SOD activity retained.

There was a significant (p<0.05) difference in the level of superoxide dismutase activity under both dark and UVA conditions. The SOD activity was found to be highest for the consortium in dark as compared to all other cell conditions. The activity of SOD was found to be significant (p<0.05) in case of individual isolates and the consortium with respect to control. This corroborates to the fact that the consortium has greater renitence as compared to the individual isolates when exposed to TiO_2_-NPs.

#### Assessment of membrane integrity by LDH Activity

Lactate dehydrogenase (LDH) is released under conditions of cell damage which is usually attributed by excessive ROS generated. The integrity of the membrane of the bacterial cells treated with TiO_2_ nanoparticle was therefore estimated by the release of cytosolic enzyme lactate dehydrogenase (Fig 3).The LDH activity under dark condition was quantified with respect to control, and was found to be 9.80 ± 0.05, 13.57 ± 0.06, 17.50 ± 0.01, 17.10 ± 0.09, 20.10± 0.08 and 20.50 ± 0.05% for the consortium, *E*. *acetylicum*, *P*. *nitroreducens*, *E*. *indicum*, *B*. *diminuta* and *B*. *flexus* respectively. The least activity in the consortium and most being in *B*. *flexus* reflects the damage in these cells, and hence their viability as seen earlier in this report.

Comparatively, LDH activity in the case of UVA treated samples was estimated to be 19.90± 0.03, 25.80 ± 0.01, 29.80 ± 0.08, 26.20 ± 0.0, 33.40 ± 0.08 and 40.0 ± 0.05% for the consortium, *E*. *acetylicum*, *P*. *nitroreducens*, *E*. *indicum*, *B*. *diminuta* and *B*. *flexus* respectively. There was a significant (p<0.05) LDH activity in the individual isolates and the consortium as compared to the control. The LDH activity was noted to be significant (p < 0.05) under both dark and UVA illuminated conditions.

### Internalization of NPs with help of Transmission Electron Microscopy (TEM)

**Fig 4:**
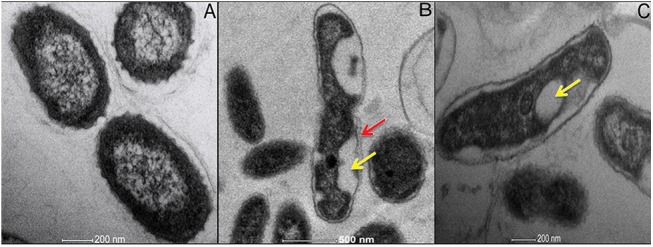
Internalization of NPs with Transmission Electron Microscopy: (A) The intact bacterial consortium, (B) Consortium interacted under dark condition showing disrupted morphology and vacuole formation, and (C) Interacted consortium cell under UVA condition depicting the vacuole formation. Notations used: Red arrow represents the distortion of the cell and yellow arrow denotes the formation of vacuoles.

To further corroborate the observations, an insight in the structural aberrations of the cells was warranted. Since the consortium of cells showed maximum resistance against the toxic effects of TiO_2_ NPs, TEM analyses was carried out with them. The cells without treatment of the NPs exhibited definite morphological features with smooth well defined and uncompromised cell wall (Fig 4A). Both dark and UVA irradiated parameters were explored, and under dark, the consortium showed patches and small clumps formation in dividing cells as shown in Fig 4B. Disruption of the cell wall under UVA condition and formation of vacuoles was observed as shown in Fig 4C.

### Analysis of EPS release with TiO_2_ NPs

**Fig 5:**
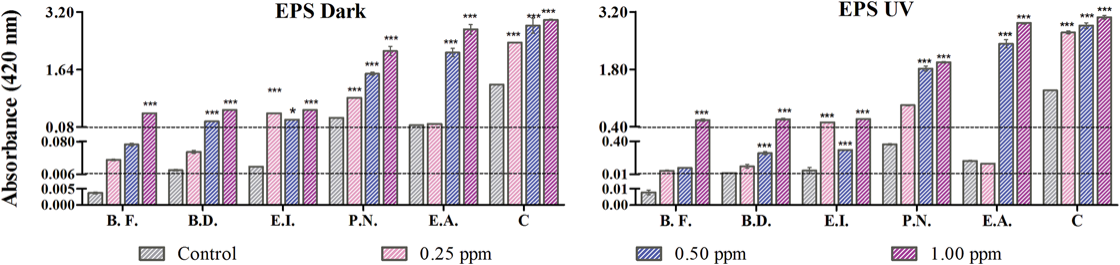
Estimation of EPS release under UVA and dark condition: EPS release at 0.25, 0.5 and 1 μg/ml of TiO_2_-NPs concentration is depicted. ‘*’ denotes the significant difference between treated cell with respect to control. The abbreviations are as follows–E.A., *Exiguobacterium acetylicum*; P.N., *Pseudomonas nitroreducens*; E.I., *Exiguobacterium indicum*; B.D., *Brevundimonas diminuta* and B.F., *Bacillus flexus*.

To evaluate the protective measures adapted by microbes as counteraction against the toxic effects sponsored by TiO_2_, an analysis of exopolysaccharides (EPS) was performed. The greater amount of EPS produced would provide better defense against the deleterious effects of NPs. Results are shown in Fig 5.

Cells under dark: The untreated cells also show EPS formation as a requisite to biofilms. The EPS produced in control had an absorbance of 1.23 ± 0.003, 0.128 ± 0.001, 0.33 ± 0.03, 0.02 ± 0.003, 0.015 ± 0.001 and 0.03 ± 0.002 A.U. and that for treated cells (with 1 μg/ml of TiO_2_ NPs) was assessed to be 2.98 ± 0.002, 2.73 ± 0.009, 2.14 ± 0.12, 0.54 ± 0.008, 0.58 ± 0.09 and 0.45 ± 0.002 A.U. for the consortium, *E*. *acetylicum*, *P*. *nitroreducens*, *E*. *indicum*, *B*. *diminuta* and *B*. *flexus* respectively. *E*. *acetylicum* showed maximum EPS secretion in dark whereas *B*. *flexus* had it lowest. Among all setups, the consortium had best response in releasing EPS, and this was comparable and followed by *E*. *acetylicum*. In all cases, comparable values were obtained for cells with an exposure of 0.5μg/ml and 1.0 μg/ml of nanoparticles. Least EPS formation was seen in cells exposed to 0.25μg/ml of TiO_2_-NPs with respect to control cells.

Under UVA condition: For the highest exposure concentrations of TiO_2_ (1 μg/ml), EPS absorbance was found to be 3.06 ± 0.04, 2.92 ± 0.02, 1.97 ± 0.02, 0.59 ± 0.005, 0.58 ± 0.02 and 0.56 ± 0.02 A.U. for the consortium, *E*. *acetylicum*, *P*. *nitroreducens*, *E*. *indicum*, *B*. *diminuta* and *B*. *flexus* respectively as compared to the control cells showing EPS having absorbance as 1.28 ± 0.09, 0.16 ± 0.02, 0.36 ± 0.08, 0.05 ± 0.03, 0.02 ± 0.002 and 0.07 ± 0.001 A.U. EPS was comparable at 0.5μg/ml and 1.0μg/ml of NPs for the consortium, *E*. *acetylicum* and *P*. *nitroreducens* only. Interestingly, EPS secretion under 0.25μg/ml and 1.00 μg/ml exposure of TiO_2_-NPs was comparable for *E*. *indicum*. The trend in EPS release was similar to what observed under dark conditions.

EPS production was found to be statistically significant (p < 0.05) as compared to the control. The EPS production in case of UVA was found to be statistically significant (p<0.05) as compared to the dark condition. Comparing dark and UVA irradiated conditions, more of EPS formation was observed under UVA. This could be related to enhanced defense mechanism under oxidative stress. EPS was found to be almost similar and maximum, in the consortium under both dark and UVA condition depicting photo-activation effect of NPs on EPS release. Under UVA, more ROS is generated which eventually when comes in contact with the cells will lead to self-defense mechanism, hence increasing the EPS release.

### Surface chemical studies of EPS from the consortium

#### FT-IR Spectral analysis

The FT-IR spectra of EPS of consortium under dark and UVA conditions exhibit characteristic functional peaks. For untreated control cells under dark conditions (S2A Fig), FT-IR peaks were observed at 3340.71 cm^-1^ corresponding to the hydroxyl group which might be due to the enhanced hydration of EPS [39]. Further, in range of 2349–2927.94 cm^-1^ that represents the CO_2_ adsorption of the amine group and also may be due to the CH_2_ vibrations of polysaccharides [40, 41]. Another peak at 1413.82 cm^-1^confirmed the presence of sugars and the ring stretching of mannose and galactose, A peak from 1535–1643 cm^-1^ corresponding to the stretching of the galactose and mannose sugars present in the saccharides, which may also represent assymetrical stretching of COO^-^ group or a bend in secondary amine group [42, 43], and finally in range of 1238–1089 cm^-1^ representing the C-N stretch of aliphatic amines.

The bands corresponding to the peaks 3516.23 cm^-1^, 3491 cm^-1^ exhibited an increase in intensity after treatment with TiO_2_-NPs under dark condition (S2B Fig). An increase in the intensity of the amine group was observed in the range of 2112.05–2922.16 -cm^-1^. The decrease in the intensity of the galactose and mannose stretching and the increase in the intensity of the COO^-^ groups represent the role of sugars. The characteristic peaks of Ti-O-Ti bands in the treated sample exemplify the presence of titania attachment to the biofilm.

The FT-IR spectra attained for control samples under UVA (S3A Fig) for the consortium cells were in broad range of 3111.18–3581.81cm^-1^ corresponding to hydroxyl group in EPS [39], at 2385.95 cm^-1^ due to the presence of amines or may be from CO_2_ adsorption[40], between 2164.13–2061.90 cm^-1^ representing the isothiocyanate and thiocyanate, at 1643 cm^-^1 identifying presence of simple sugars as mannose or galactose, in the range of 1408. 04–1494.83 cm^-1^ representing the stretching of COO^-^ group [41], and in wide band present between 1010.70–1145.72 cm^-1^ represents ester linkage of uronic acids [44]. The peaks present in the range of 700–800 cm^-1^ may attribute to the presence of polysaccharides in EPS [45].

The treatment of consortium with nanoparticles under UVA conditions (S3B Fig) exhibited a peak shift from 3026.31 to 3614.60 cm^-1^ that represents the change in the hydroxyl groups present in the EPS. The increase in peak intensity at 2387.87 cm^-1^ represents the variation in the amines. A broad peak from 2038.76–2281.79 cm^-1^ represents the functional group changes in isothiocyanate and thiocyanate. The increase in the intensity from 1502.55–1660.71 cm^-1^ signifies the presence of sugar in the EPS. The presence of content of uronic acid in the EPS has increased to 1180.44 cm^-1^ and also an increased peak was observed at 785.03 cm^-1^ denotes the presence of polysaccharides. A distortion in the peaks was observed at 500–600 cm^-1^ reveals the presence of titania attached to the biofilm.

The comparative study of UVA and dark signifies that the presence of carbohydrates in the form of simple sugars and polysaccharides that may contribute to the components of carbohydrate in EPS. The presence of amines and the change in the intensity denotes the significance of proteins in EPS.

#### Zeta Potential measurement

An estimate of zeta potential renders the stability of the particle in suspension. Lower the potential, the more unstable is the particle, and therefore will collide together to form bigger aggregates. The zeta potential of EPS from the consortium was therefore assessed. Under dark condition, it was found to be -10 ± 0.8 mV for EPS without TiO_2_-NPs exposure. The zeta potential measurement of treated cells under dark condition was estimated to be—4.3 ± 0.5 mV. The substantial surface potential on the control consortium cells under UVA condition was estimated to be -5.8 mV and the potential on the treated cell was found to be -1.1mV. The values obtained for zeta potential of cells favors cell adhesion and formation of EPS. Clearly, TiO_2_-NPs treated cells are more prone to adhere. Moreover, UVA irradiation favors the process to a greater extent when compared to dark conditions

### Biofilm formation

Biofilms are usually defined as multicellular components of microorganisms domiciling in hydrated high complex molecular weight complex known extra polysaccharides (EPS) [46]. According to a previous report biofilm mass grows with the interaction with TiO_2-_NPs in LM media with 24 h of incubation [16]. In this study, the biofilm formation capacity of all the individual isolates and their consortium control and treated cells was assessed.

**Fig 6:**
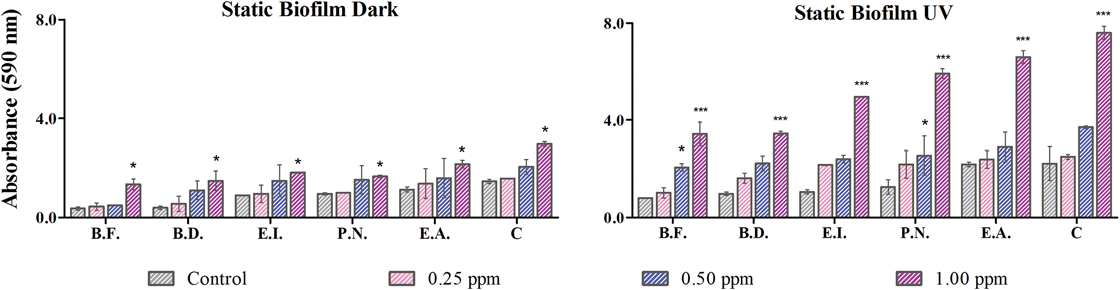
Biofilm aggregation: Assessment of biofilm formation under dark and UVA condition at 0.25, 0.5 and 1 μg/ml of TiO_2_ NPs. ‘*’ represents significant difference of treated cells with respect to control (n = 3). The abbreviations are as follows–E.A., *Exiguobacterium acetylicum*; P.N., *Pseudomonas nitroreducens*; E.I., *Exiguobacterium indicum*; B.D., *Brevundimonas diminuta* and B.F., *Bacillus flexus*.

The formation of biofilm under dark condition (Fig 6) in the case of control cells gave an absorbance of 1.46 ± 0.09, 1.14 ± 0.105, 0.965 ± 0.04, 0.901 ± 0.009, 0.4 ± 0.06 and 0.37 ± 0.06 A.U. which further enhanced in the treated cells and was assessed to be 2.98 ± 0.09, 2.17 ± 0.16, 1.67 ± 0.05, 1.82 ± 0.02, 1.5 ± 0.38 and 1.35 ± 0.21 A.U. for the consortium, *E*.*acetylicum*, *P*. *nitroreducens*, *E*. *indicum*, *B*. *diminuta* and *B*. *flexus* respectively.

The biofilm formation for the control cells was evaluated to give absorbance of 2.22 ± 0.01, 2.19 ± 0.69, 1.26 ± 0.06, 1.06 ± 0.09, 0.98 ± 0.30 and 0.8 ± 0.09 A.U. The capacity to form biofilm was further enhanced when treated with 1 μg/ml of TiO_2_-NPs under UVA condition (Fig 6) and was estimated to be 7.6 ± 0.47, 6.6 ± 0.27, 5.92 ± 0.08, 4.96 ± 0.268, 3.45 ± 0.19 and 3.432 ± 0.01 A.U. in the same order as for dark.

The difference of biofilm formation between the individual isolates and the consortium was found to be significant (p <0.05) as compared to the control. At the highest exposure concentration (1 μg/ml of TiO_2_ NPs) the difference in the biofilm formation was found to be significant (p<0.05) under both UVA and dark condition. Biofilm formation was seen to be much higher in consortium and especially the ones irradiated with UVA. A plausible explanation to such observation could be the higher extent of release of EPS with a very low zeta potential under UVA irradiation, therefore facilitating adhesion.

#### SEM analysis of biofilm formation

**Fig 7:**
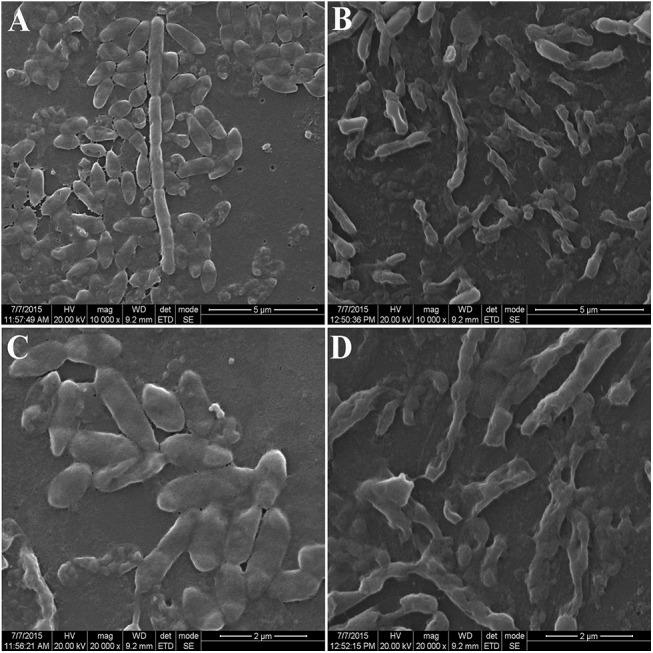
Scanning electron microscopy of biofilm formation: (A) Biofilm formation of control consortium cells under dark condition (B) Interacted biofilm formation at 1 μg/ml TiO_2_-NPs under dark condition (C) Biofilm formation of control consortium under UVA condition (D) Micrograph representing the treated biofilm under UVA condition.

A visual perception of the control and treated cell surface is usually required for a justified exploration. Hence, the architecture of the biofilm of the consortium with and without treatment of TiO_2_ nanoparticle was analysed with SEM. After 24h of incubation, the control consortium depicted smooth bacterial cells under dark (Fig 7A) and UVA conditions (Fig 7C).The nanoparticle treated (at 1 μg/ml) biofilm under dark and UVA conditions (Fig 7B and 7D) exhibited perforated and ruptured morphology. Cells exposed in UVA were more prone to biofilm formation, and the same can be observed in SEM images. The control cells also formed biofilm and hence shows agglomeration, which was much pronounced in TiO_2_-NP treated consortium. Although, a slight adhesion was also seen in control cells in dark, but individual cells were fairly distinct. The presence of titanium on the treated biofilm was observed by EDX analysis (S4 Fig) element is due to the reason that the samples were coated on glass slides and the presence of Au element is due to the gold sputtering done prior to the analysis to neutralize the charge of the samples.

## Discussion

The physical characterization of TiO_2_-NPs with varying concentrations in the test medium, as used in the study, was a prerequisite to infer the toxic effects imparted by these NPs on the bacterial isolates and their consortium. Since the medium is waste water, it is obvious that numerous chemicals, high concentrations of cations and surfactants would attribute to its nature, which we have seen to be unfavorable for TiO_2_-NPs stability. An immediate aggregation is observed as soon as the particles are dispersed in waste water, rendering particles of more than 500 nm in size (Fig 1). This aggregation reduces the specific surface area of NPs and therefore will affect the intensity of their toxicity[47]. The increase in particle size indicates a loss in the charge of nanoparticles, which could be an outcome of the interactions with different ions present in the media. This is supported by the zeta potential values over 12h of time, where we observed a successive decrease (results not shown). The natural organic matter (NOM) present in the waste water may agglomerate with the introduction of nanoparticles with respect to time [48]. The nanoparticles stabilized by organic matter holds the ability to interact with the negatively charged surface of bacteria and successively internalize in the cells [49], also noted in TEM and EDX analysis (S4 Fig). The aggregated NPs, as a result of co occurring solute ions in the medium, are usually unable to pass through the cell membrane, and therefore would show their toxic effects through generating ROS either via photo-activation or energy transfer from activated carbon centers of organic molecules[50].

This study aims to prove the ability of consortium to more efficiently counteract the toxic effects exerted by TiO_2_-NPs. Positively; the consortium had less observed deleterious effects. There are various reports on cytotoxic impacts of bacterial interaction with TiO_2_-NPs under different conditions. According to a previous report, *E*. *coli* interacted with TiO_2_-NPs by utilizing artificial water as a media. The survival rate of *E*. *coli* was approximated to be 60% after 2 h of exposure to UVA [51]. There was a 75% reduction in viability of *E*. *coli* cells, when treated with 100 μg/ml of TiO_2_-NPs and there was no difference in the viability under dark condition [52]. The role of superoxide and hydroxyl radicals as ROS has been attributed to such toxic effects. *Listeria monocytogenes* after an exposure of 24 h with TiO_2_-NPs revealed no significant change (considering p<0.05 as significant) in the viability [16]. A study with *E*. *coli* interacted with 8 μg/ml of TiO_2_ nanoparticle for a period of 60 min incubation in phosphate buffer saline as a medium exhibited 19% reduction in viability [53]. However under UVA preillumination, *Bacillus anthracis* when interacted with TiO_2_-NPs exhibited a synergistic toxic effect [54]. Exposure to UVA or redox active molecules (ions and radicals) in the media activates TiO_2_-NPs and makes it unstable, as noted in the current study, which therefore leads to its interaction with the microbial surface and hence causing toxicity. The toxic effects are usually observed under photo-activated TiO_2_-NPs, therefore, under dark, inhibitions are rarely reported. In our study though, the medium used is treated waste water from sewage treatment plant, which already contains numerous redox active molecules (*viz*. free radicals and ions). These molecules can well bring redox change in the NPs and affect to produce ROS. TiO_2_-NPs when in contact with bacterial cell membrane shows its activity under dark condition [12, 55]. Unlike the majority of prior toxicity studies, the current study focuses on the viability of bacterial colonies in waste water because of their importance in sewage treatment. The viability results depict the robust nature of consortium as compared to single isolates, which is favorable for sewage treatment.

According to a prior report, increase in the concentration of TiO_2_-NPs leads to the greater oxidative stress that may be responsible for decline in cellular viability [56]. TiO_2_ is known to be activated with UVA irradiation that leads to ROS generation and subsequently causes observed for the same. There are some reports manifesting ROS generation under dark condition as the attachment of nanoparticles to the cell surface. Further the attachment induces the release of carbon centred free radicals that aggravate the oxidative damage to the cell wall of bacteria [50]. According to Burello and Worth, ROS generation from UV excited oxides of NPs is feasible when their conduction band lies in the range of biological redox potential [57], which they have shown to be favorable for TiO_2_. The elevation in ROS generation observed for cells treated in dark as compared to UVA could be attributed to electron transfers from redox molecules in the medium to TiO_2_-NPs thereby activating them and hence leading to cell damage. The degradation of complex organic and inorganic compounds in waste water requires the presence of ROS. This can be considered advantageous with respect to digestion of colloids but their (ROS) high concentration also leads to cell damage. Over time, ROS generation due to TiO_2_-NPs would not be substantially effected, which is deleterious for cell viability, since a major contribution is that of ROS generated with effect to the viable cells. In the current study the generation of ROS was low in case of consortium as compared to the individual isolates. This clearly confirms that viability of consortium is more prominent as compared to single isolates and thereby ascertains the potent role of consortium in natural environment of waste water treatment process.

Higher SOD activity relates to higher ability of the cells to dismutase superoxide, and hence the cells possess greater chance to detoxify the effects of superoxide. Since, ROS generation is continuous, owing to the presence of NPs; a constant defense is needed to protect the cells. Denaturation of suppressed / inactive SOD enzyme weakens the defense system of the bacterial cells and eventually degrades other functionally active enzymes. Consequently, more ROS will accumulate and cell organelles exposed to them will be rendered dysfunctional leading to collapse of complete cellular machinery. The increased SOD activity exhibited by consortium is a synergistic response from multiple species. The mechanism or role of consortium in regulating a function involves multiple control points. Although every individual species in the consortium have the capacity to perform multiple functions, it is with varied efficiency. The species more potent of controlling a pathway or cellular mechanism is usually assigned to monitor that function, similar to division of labor[58]. Under these conditions, since specific functional roles are distributed among different species, more resources in form of energy can be saved or utilized for reduced number of functions. The role assigned in such an environmental setup to a (group of) species is therefore performed more efficiently than in its individual existence, since the energy is directed specifically for a set of cellular functions as the rest is being taken care by other species efficient in other functions. In the present study, *Exiguobacterium acetylicum* and *Pseudomonas nitroreducens* have higher capacity to produce SOD enzyme and thus plays a vital role for defending the oxidative stress imparted by TiO_2_-NPs. Similar observation has been reported by Robertson *et*. *al*. and Leung *et*. *al*. [59, 60]. According to their report, interacted cells depicted that SOD acts a defensive system for the bacteria.

According to the previous reports, TiO_2_-NPs causes membrane permeabilization and distorted membrane integrity [11, 61]. Loss in membrane integrity due to these NPs is a consequence of either their attachment to cells or due to ROS generation, and consecutively changing the biological redox of their surface (membrane) which thereby sequentially induces disintegration of its structure. Attachment of TiO_2_-NPs to bacterial cell surface requires the release of Ti^4+^ ions since both bacterial cell and the nanoparticle is negatively charged [62]. However, chemical dissolution of TiO_2_-NPs is very low and therefore, we cannot assume a lot of Ti^4+^ attachment to the surface. TiO_2_ is thermodynamically insoluble at experimental conditions. The presence of Ti^4+^ in waste water usually occurs in solid phases and not in ionic forms. Thus the toxic effect of NPs is due to the factors as ROS, LDH, adsorption of particle on surface. This suggests, membrane damage is majorly a resultant of ROS released in the medium. In either of the case, eventually the ROS generated interacts with polyunsaturated phospholipid component of bacterial membrane and hence leads to its peroxidation. Due to this peroxidation, the cell membrane loses its integrity leading to release of LDH in the medium. A relatively low LDH activity was ensured in consortium as compared to single isolates this confirms the less oxidative stress in consortium as compared to individual isolates. Nanoparticle attachment to bacterial surface may also lead to the enhanced intracellular ROS. This increase in ROS further stamps down the metabolic balance and homeostastis of bacterial cell. According to Szczupak et al [63] the penetration and adsorption of NPs in the cell may lead to the inactivation of proteins in the bacterial cell membrane that eventually lead to cell death. Earlier reports [64] also exemplifies distorted morphology and disorganized membrane of *E*. *coli* when treated with TiO_2_. There are few reports depicting the mechanism of modification of bacterial cell membrane through TEM studies and hence describing the release of intracellular K^+^ ions and proteins from the damaged cell membrane[65]. Since internalization is possible only upon attachment of NPs to cell surface, and because both of them are negatively charged, we would not observe significant uptake normally. But, increased LDH release, abbrations of cell seen in TEM images and amount of damaged cells observed in CLSM images suggest substantial damage to cells even without internalization.

EPS plays an important role in the formation of biofilm and protects the cells from dehydration. Tsuneda *et*. *al*. [35] suggested that polymeric interactions due to EPS release enhance the cell adhesion. Since EPS facilitates the attachment of TiO_2_-NPs on its surface [18], majority of these nanoparticles will adhere to EPS thereby reducing the amount of free nanoparticle to attack the bacterial cells in the medium. ROS generated at the surface of EPS needs to diffuse through to get an access to the bacterial cells to cause any damage. Although there will be sufficient damage to the bacterial colonies attached near the surface of EPS, since they would first come in contact with the deleterious ROS, the cells deep inside will be protected from any degeneration. The capsular EPS provides a defense against the attachment of nanoparticle and ROS activity directly to the bacterial cells thereby abridging membrane integrity losses. The release of EPS in case of consortium is higher as compared to the individual isolates. EPS is made up of polymeric matrix that consists of forces like electrostatic and van der Waals; this matrix traps the interacting nanoparticle and hence acts as a defense system for the microbes. The amine groups on the surface of EPS are positively charged. Interaction of negatively charged TiO_2_-NPs to EPS surface is feasible only through its attachment to the positively charged amines. The high intensity shown by these amine groups in FT-IR spectra indicates their abundance on EPS surface and hence provides sufficient surface for the NPs to interact. The bend observed in secondary amine (1535–1643 cm^-1^) is possibly due to this attachment.

With an increase in NPs concentration, ROS level and therefore toxicity level is bound to increase. To counter these toxic effects, cells naturally would form an association, which we see as biofilm formation. We have also observed an enhanced release in EPS which itself is due to a defense mechanism of bacterial colonies. EPS release provides the necessary environment for biofilm formation, and with an increase in EPS release, we expect more biofilm, as corroborated with the results. The biofilm morphology is greatly affected by EPS secretion. More the amount of EPS released, bigger the biofilm formation. Even through SEM, we noticed that maximum biofilm formation was seen in UVA treated cells. TiO_2_-NPs when adhere to the cell surface, and generate ROS, the cells encounter damage. As a result of that, defense mechanisms are activated; EPS release being a part of it. At the end of 24 h, most of the nanoparticles are devoid of access to bacterial cell surface since they are held back on the structural framework of EPS. This gives an opportunity to form larger biofilms, whose cells are intermittently exposed to nanoparticles, which bypass the EPS framework. These nanoparticles, after crossing the EPS matrix barrier, might adhere to the cell surface, re-initiating the cascade releasing more of EPS and allowing even bigger biofilms. Perhaps, that is the reason why we observed better viability in UVA exposed cells rather than the ones in dark.

**Fig 8:**
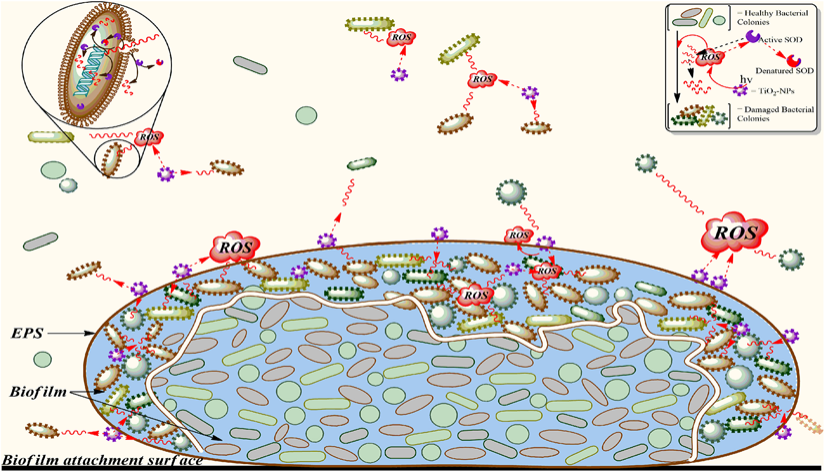
Interaction of TiO_2_-NPs with consortium: Schematic representation of a cascade of interactions between bacterial cells and energy (hʋ) activated TiO_2_-NPs and its effects are shown. A result of NPs activation is ROS (shown as red cloud) which diffuses (red wavy lines) through the medium, EPS surface and biofilm. Intracellular ROS generation for cellular function is not considered and hence not shown. Different species (shown with different structure and color) of healthy bacterial cells (smooth surface) when acted upon by ROS, confronts damage and loses its integrity (shown as rough surface). Inside the EPS surface, the upper layer of cells block the diffusion of ROS to the inner core (separated with a wavy doubled line) and the stabilized biofilm effectively defends deleterious effects of ROS. The ROS rendered futile due to the action of SOD is shown as broken wavy red lines. Some of the SOD enzyme also gets denatured (patchy red-purple enzyme) while counteracting the ROS effects.

A schematic representation of the micro-environment depicting the interactions of TiO_2_-NPs, generated ROS and bacterial cell colonies free in the medium as well as inbound in the EPS matrix as biofilm is shown in Fig 8. The amount of TiO_2_-NPs shown on the surface of EPS is much higher than in free medium. The activation of NPs due to energy transfer leads to ROS generation which disperses in the media and causes damage to cells. Futility of ROS depends on the activity of SOD, which is enhanced in consortia and in the biofilm formed. EPS, acting as a protective layer, attaches most of the NPs on its surface, but the remaining ones in medium bring substantial damage to single colonies. Cell damage leading to disruption of membrane eventually releases LDH in the medium. The effective toxicity is greatly reduced due to the formation of consortial biofilm inside the EPS, since most of the cells are far from exposure to deleterious species in the medium.

## Conclusion

The toxic effects of TiO_2_ NPs on single bacterial species in waste water are very well exemplified at several instances including this manuscript, but the advantages of consortium to combat such deleterious effects has been explored here in detail. The present study provides a holistic understanding on the behavior and interactions of individual isolates and the consortium in waste water medium in presence of TiO_2_-NPs at low concentrations under dark and UVA condition. The cytotoxic effects in relation to oxidative stress generation were studied as the major endpoints to compare the differences between the individual isolates and the consortium of cells. As observed from the viability study, consortium was found to be more resistant to the toxic effects of TiO_2_-NPs as compared to individual bacterial isolates. Further ROS generation and SOD activity substantiates the viability results. The role of EPS release and consequent biofilm formation as possible defense mechanism was compared between the individual isolates and their consortium. Even below 1 μg/ml TiO_2_-NPs concentrations, enhanced EPS release as well as biofilm formation in the consortium compared to single isolates was noted. Consistently, the consortium of cells proved to have better abilities in resisting the toxic effects of TiO_2_-NPs. In waste water treatment plant therefore, the activity of consortium should be checked and analyzed for subverting the toxic effects of NPs (TiO_2_). The future prospects could be the screening of bacterial species that has an inherent pronounced capability of EPS production and that shows no response against phototoxic effect of TiO2 NPs and are equally capable of waste water treatment.

## Supporting Information

S1 Fig CLSM images of the consortium: (A) Cells appearing green shows the presence of live cells (B) red colour denotes dead cells. (TIF)

S2 Fig FT-IR spectra for EPS of the consortium under dark condition: (A) Represents the FT-IR peaks of EPS control (B) Treated EPS with 1 μg/ml of TiO2 NPs under dark condition (TIF)

S3 Fig FT-IR spectra for EPS of the consortium under UVA exposure: (A) Represents the FT-IR peaks of EPS control (B) Treated EPS with 1 μg/ml of TiO_2_ NPs under UV condition (TIF)

S4 Fig EDX analysis of the biofilm from the consortium: EDX analysis depicting presence of elemental titanium in the biofilm (TIF)
