# Supplementary figures and images for: Decreased Phototoxic Effects of TiO₂ Nanoparticles in Consortium of Bacterial Isolates from Domestic Waste Water
Source: PLoS One. 2015 Oct 23;10(10):e0141301. doi: 10.1371/journal.pone.0141301 (PMC4619802; doi:10.1371/journal.pone.0141301)

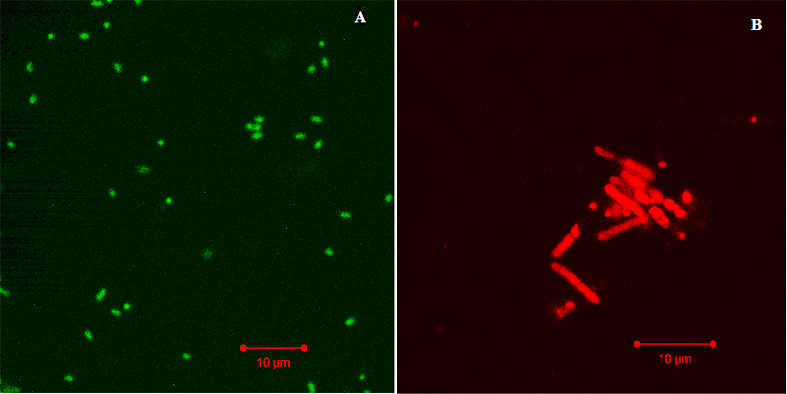

Supplement: S1 Fig — (TIF) [file pone.0141301.s001.tif]

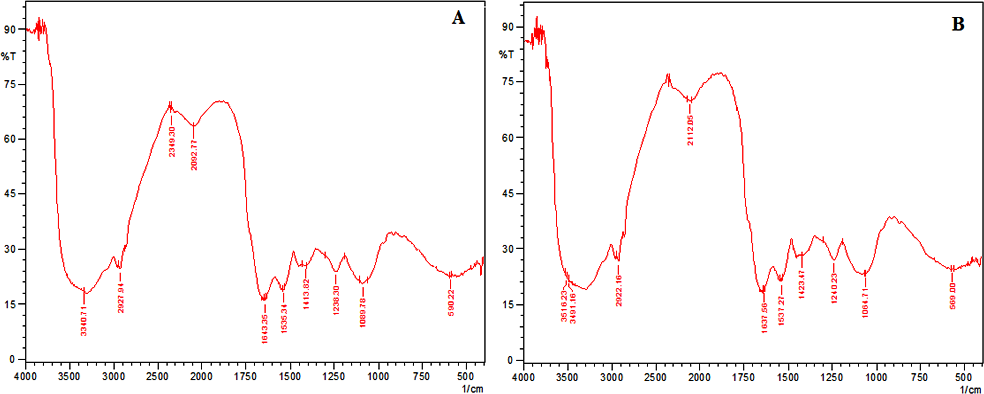

Supplement: S2 Fig — (TIF) [file pone.0141301.s002.tif]

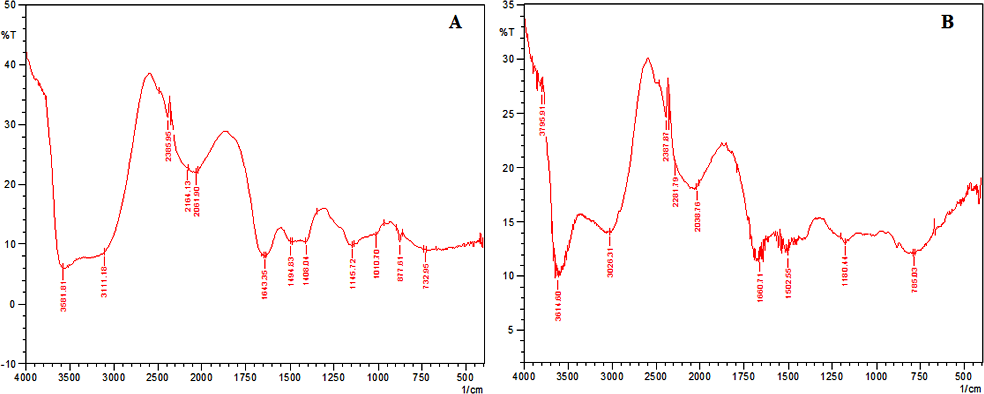

Supplement: S3 Fig — (TIF) [file pone.0141301.s003.tif]

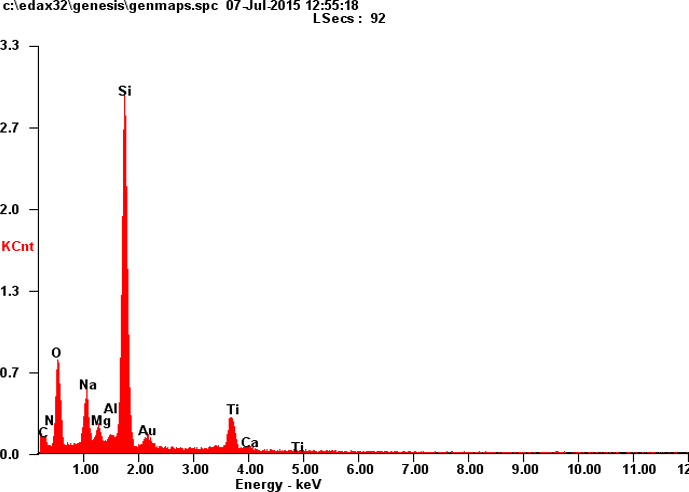

Supplement: S4 Fig — (TIF) [file pone.0141301.s004.tif]
